# Correction to: Systematic analyses reveal long non-coding RNA (PTAF)-mediated promotion of EMT and invasion-metastasis in serous ovarian cancer

**DOI:** 10.1186/s12943-020-01296-1

**Published:** 2020-12-29

**Authors:** Haihai Liang, Xiaoguang Zhao, Chengyu Wang, Jian Sun, Yingzhun Chen, Guoyuan Wang, Lei Fang, Rui Yang, Mengxue Yu, Yunyan Gu, Hongli Shan

**Affiliations:** 1 Department of Pharmacology (State-Province Key Laboratories of Biomedicine-Pharmaceutics of China, Key Laboratory of Cardiovascular Research, Ministry of Education), College of Pharmacy, Harbin Medical University, Translational Medicine Research and Cooperation Center of Northern China, Heilongjiang Academy of Medical Sciences, Harbin, 150081 China; 2 Department of Systems Biology, College of Bioinformatics Science and Technology, Harbin Medical University, Harbin, 150001 China; 3 Department of Pathology of The Second Affiliated Hospital, Harbin Medical University, Harbin, 150081 China; 4 Department of Pathology of The First Affiliated Hospital, Harbin Medical University, Harbin, 150081 China; 5 Department of Obstetrics and Gynecology, The Second Affiliated Hospital of Harbin Medical University, Harbin, 150081 China; 6 Department of Systems Biology, College of Bioinformatics Science and Technology, Harbin Medical University, Harbin, 150086 China; 7 Training Center for Students Innovation and Entrepreneurship Education, Harbin Medical University, Harbin, 150086 China

**Correction to: Mol Cancer 17, 96 (2018)**

**https://doi.org/10.1186/s12943-018-0844-7**

Following the publication of the original paper [1], the authors found an inter-duplication in Fig. 3d and a misplacement in Fig. 6e and herein make corrections of these errors. First, the “Wound-healing Assay” image of “Ctrl of 24h in A2780 cells” in Fig. 3d contained an inter-duplication with the image of “NC of 24h in A2780 cells” in Fig. 2d. Second, the images in Fig. 6b for the “PTAF+miR-25a” group, in Fig. 6c for “pcDNA3.1” and in Fig. 6f for “TGF-β1 + sh-Scramble” were mistakenly presented in Fig. 6e. Our careful double-check of the original images clarified that this mistake was caused by an unintentional overlay of the images for Fig. 6b, Fig. 6c and Fig. 6f on top of the correct images for Fig. 6e during figure preparation. These mistakes have now been corrected in the revised version of Fig. 3d and Fig. 6e. The corrections do not affect the original findings and conclusions of the article. The authors apologize for any inconvenience caused by the unintentional mistakes.

Corrected figures are provided below.

**Fig. 3:**
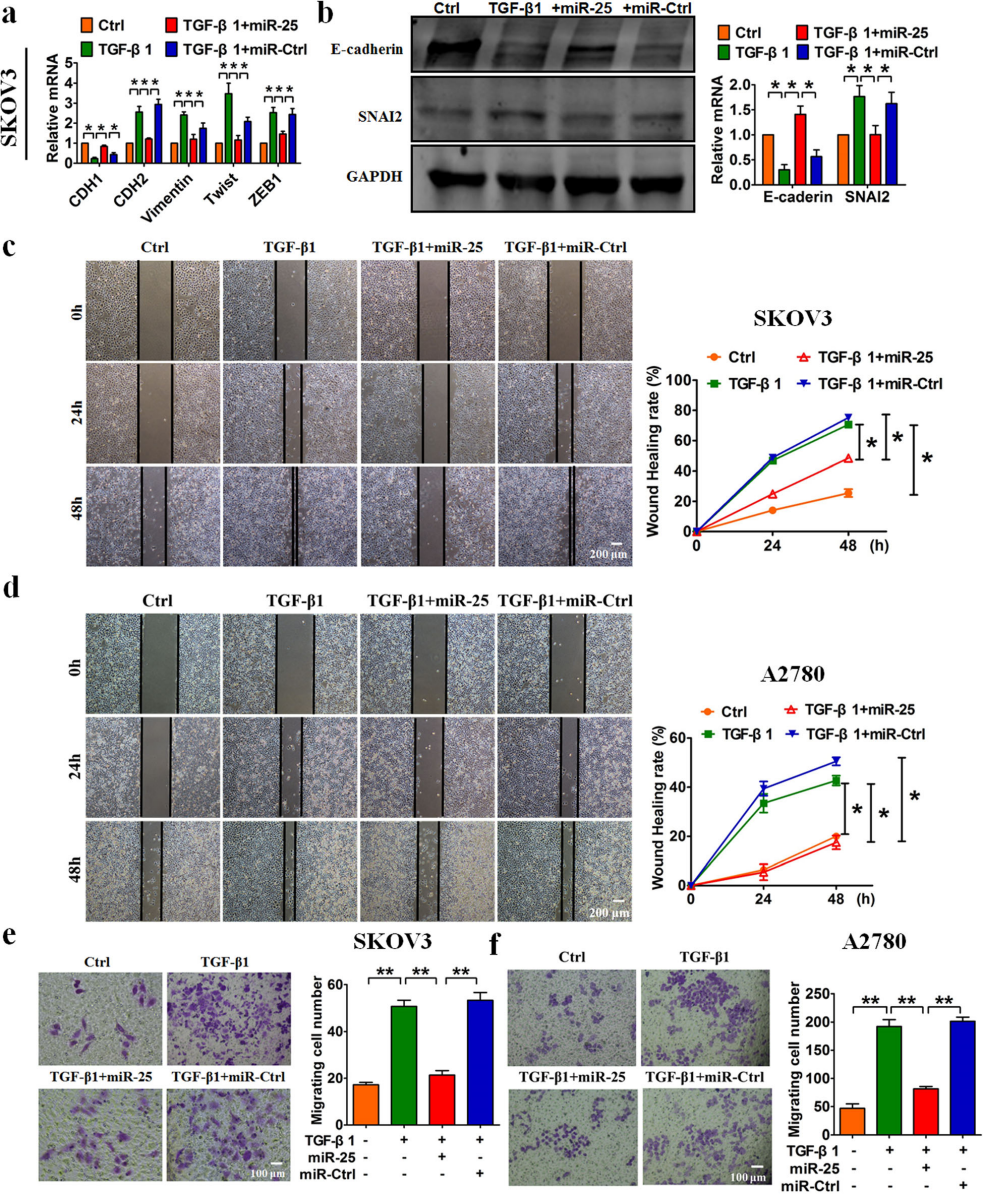
Forced expression of miR-25 blunts TGF-β1-induced EMT and migration in OvCa cells. qRT-PCR (**a**) and western blot (**b**) analyses showed the inhibitory effect of miR-25 on EMT in SKOV3 cells treated with TGF-β1. A wound-healing assay displayed the inhibitory effects of miR-25 on TGF-β1-induced migration in SKOV3 cells (**c**) and in A2780 cells (**d**). A migration assay showed that miR-25 attenuated TGF-β1-induced migration in SKOV3 cells (**e**) and A2780 cells (**f**). *n* = 5 independent experiments. *P < 0.05, **P < 0.01

**Fig. 6:**
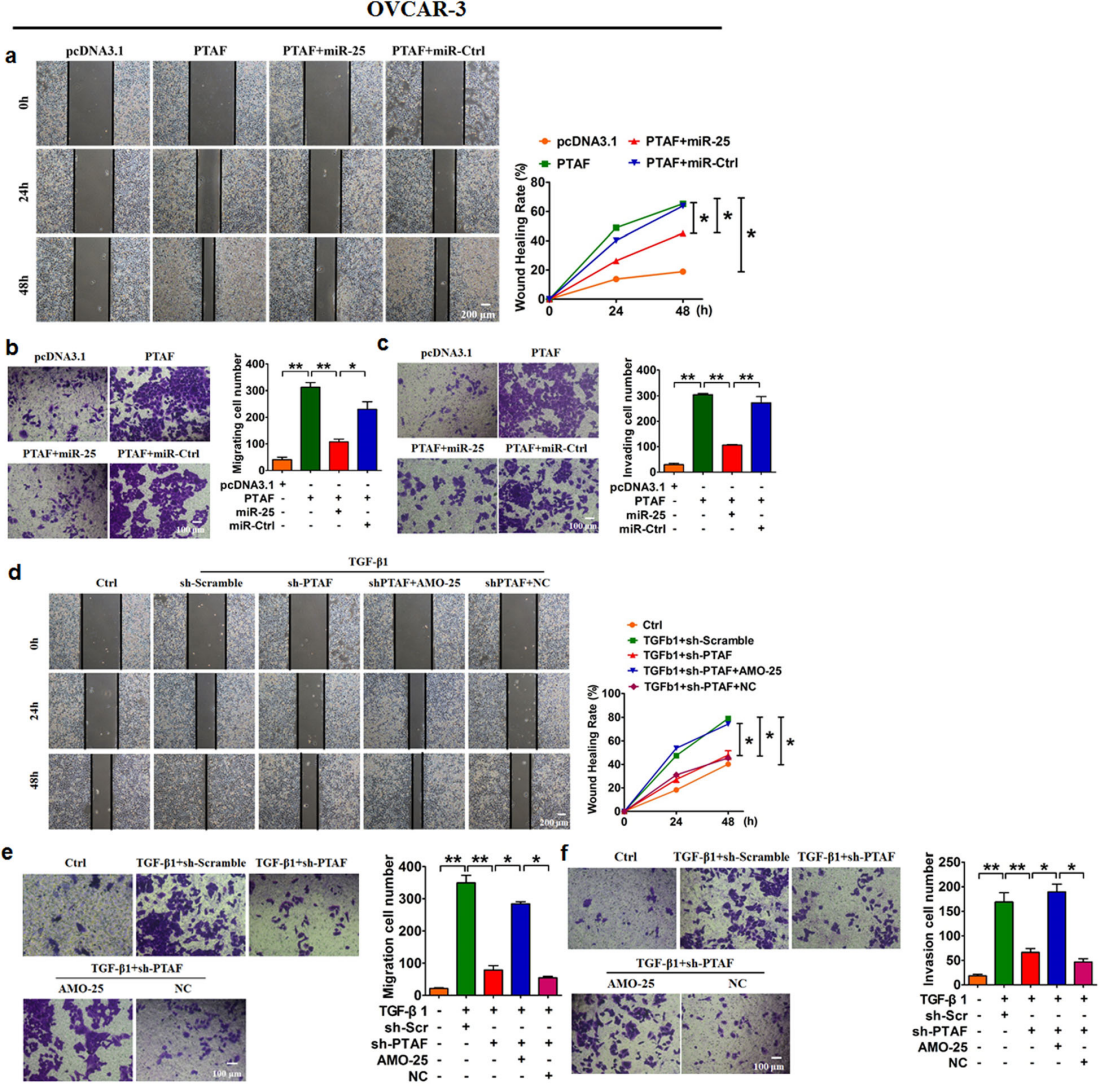
PTAF overexpression leads to OVCAR-3 cell migration and invasion by regulating miR-25. Wound-healing **a** and migration assays **b** showed that PTAF promoted OVCAR-3 cell migration, which was inhibited by forced expression of miR-25. *n* = 5 independent experiments. *P < 0.05*,* **P < 0.01. **c** A Transwell invasion assay showed that PTAF promoted OVCAR-3 cell invasion by regulating miR-25. *n* = 5 independent experiments. **P < 0.01. Wound-healing **d** and migration assays **e** showed that silencing of PTAF inhibited TGF-β1-driven migration in OVCAR-3 cells, which was abated by miR-25 knockdown. *n* = 5 independent experiments. *P < 0.05, **P < 0.01. **f** A Transwell invasion assay showed that knockdown of PTAF inhibited TGF-β1-induced OVCAR-3 cell invasion. *n* = 5 independent experiments. *P < 0.05, **P < 0.01
